# The Effect of Different Non-Metallic Inclusions on the Machinability of Steels

**DOI:** 10.3390/ma8020751

**Published:** 2015-02-16

**Authors:** Niclas Ånmark, Andrey Karasev, Pär Göran Jönsson

**Affiliations:** 1 Department of Materials Science and Engineering, KTH Royal Institute of Technology, Stockholm SE-100-44, Sweden; E-Mails: karasev@kth.se (A.K.); parj@kth.se (P.G.J.); 2 Department of Materials and Manufacturing, Swerea KIMAB, Kista SE-164-40, Sweden

**Keywords:** inclusions, machinability, steel

## Abstract

Considerable research has been conducted over recent decades on the role of non-metallic inclusions and their link to the machinability of different steels. The present work reviews the mechanisms of steel fractures during different mechanical machining operations and the behavior of various non-metallic inclusions in a cutting zone. More specifically, the effects of composition, size, number and morphology of inclusions on machinability factors (such as cutting tool wear, power consumption, *etc*.) are discussed and summarized. Finally, some methods for modification of non-metallic inclusions in the liquid steel are considered to obtain a desired balance between mechanical properties and machinability of various steel grades.

## 1. Introduction

Advances in steelmaking during the last six decades have resulted in steel grades with very low level of impurities. In recent years, new “clean and ultra-clean” steels have been developed and commercialized by steel producers around the world, thereby responding to the current and future market demands of steel having significantly improved mechanical properties (e.g., fatigue strength and impact toughness) and an improved corrosion resistance. These steels may have an extremely low content of oxygen (<10 ppm O) and sulfur (<10 ppm S). The driving force behind these advances has been to enable new steels that can tolerate highly demanding applications e.g., transmission components for the automotive industry, and construction parts and tubes for aggressive and corrosive environments.

Although today’s high-cleanliness steels have excellent mechanical properties and/or corrosion resistance, these advances in functional properties have come at the expense of more difficult chip breaking and in some cases a considerably reduced tool life in machining operations. Thoors *et al.* [1] has reported on the combined effect of hardness, steel composition and sulfide content on the ultimate tensile strength and machinability in turning of ball-bearing steel (~0.06% S) and two steels of a quench and tempering grade SS2541 (0.019%–0.20% S). The bearing steel contained fewer sulfides and had a lower hardness (215 HB) than the quench and tempering steels (about 284 HB). It resulted in an inferior ultimate tensile strength and tool wear. More accurate comparisons regarding the combined effect of inclusions on functional properties can be obtained by tests of a single steel group. Monnot *et al.* showed that the machinability of bearing steels was improved at the expense of rotating bending fatigue performance [2]. Similar balance of cutting tool life (machinability) and corrosion resistance was found for super-duplex stainless steel [3].

**Figure 1:**
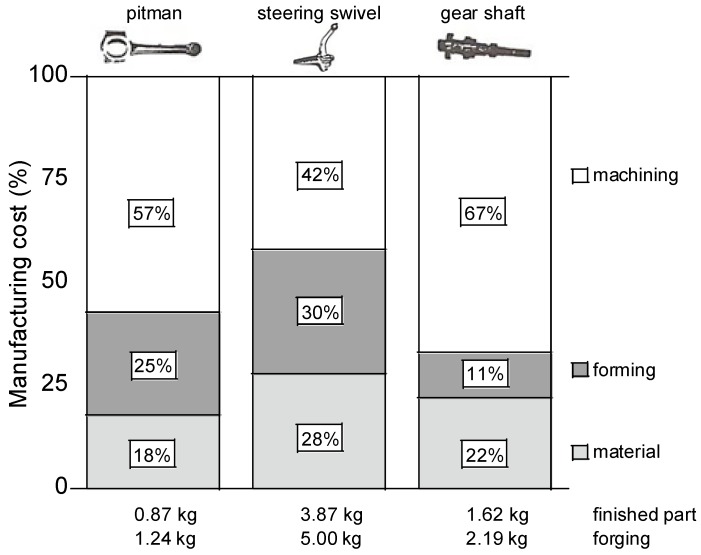
More than 40% of the total manufacturing cost in component manufacturing comes from different machining operations [4].

Machining of high-cleanliness steels is, in general, associated with a high energy consumption, an increased cutting tool wear, and high manufacturing costs. It has been estimated that more than 40% of the total manufacturing cost to produce an automotive component comes from different machining operations [4,5] (see Figure 1). Therefore, the remaining issue is assessed as to optimize today’s steel grades with respect to the combined machinability and performance requirements. In conclusion, non-metallic inclusions are to some extent necessary for a proper machinability performance. However, the content and the characteristics of non-metallic inclusions must still ensure that high performance properties of the steel can be obtained.

**Figure 2:**
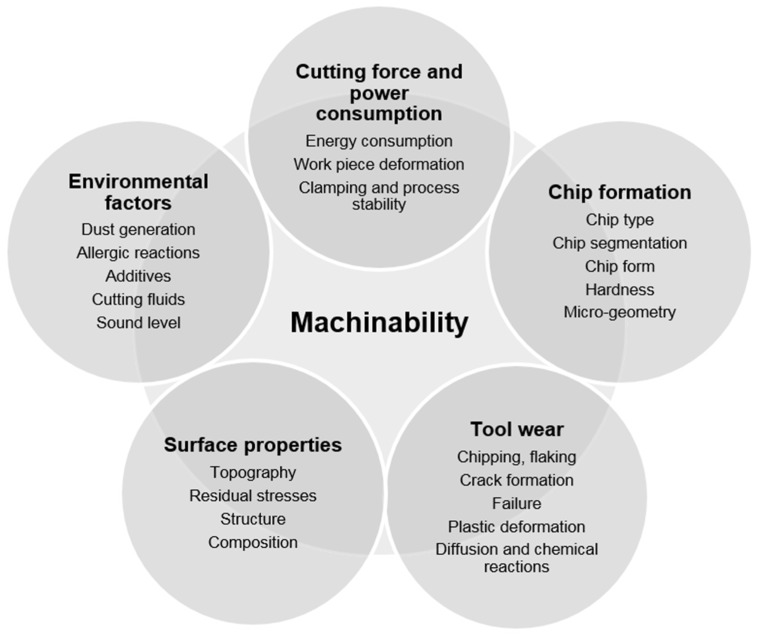
A schematic illustration of the overall parameters that is included in the complex machinability concept.

Machinability itself is a complex concept and includes a wide range of parameters and factors. The complexity lies within the fact that each machining operator interprets machinability differently. Furthermore, for a number of operations a specific machinability criterion is used. Initial considerations usually include a component type, a component size, the number of components to manufacture and a machining operation. However, the limitations are often decided by the customers’ demands on the properties and surface quality of the component, which in turn governs the machining process, the cutting tool to use and the appropriate cutting data. From a wider range of perspective, a machinability measure should indicate a materials’ general machining ability and not only for a specific product or process. The machinability concept can therefore be divided into five general machinability parameters, namely the cutting force and power consumption, chip formation, cutting tool wear, surface properties of machined work piece and environmental factors [6] (Figure 2).

**Figure 3:**
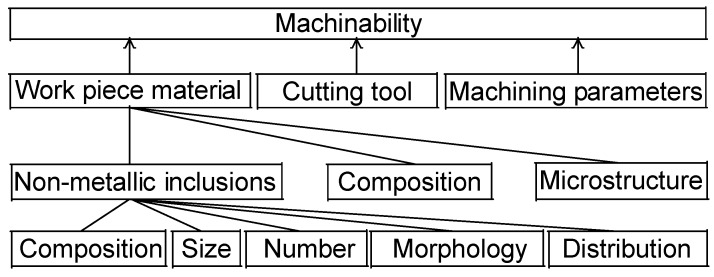
A schematic illustration of how machinability is connected to underlying factors and parameters including properties of work pieces, cutting tools and the machining conditions.

In this work, machinability is approached as a concept consisting of three main factors, namely properties of the work piece material, properties of the cutting tool and machining parameters (Figure 3). The parameters that contribute to the different properties of work piece materials are non-metallic inclusions (composition, size, number, morphology and distribution), composition and microstructure.

Although previous research has to some extent described the link between machinability and inclusion characteristics, it is now time to summarize in a wider perspective and from a metallurgical point of view. Therefore, this paper reviews and summarizes the effect of different non-metallic inclusions on the machinability of various steels. In addition, possible modifications of non-metallic inclusions to obtain an improved machinability but maintained material properties are discussed.

## 2. Metal Fracture during Machining

### 2.1. Different Techniques of Mechanical Machining

**Figure 4:**
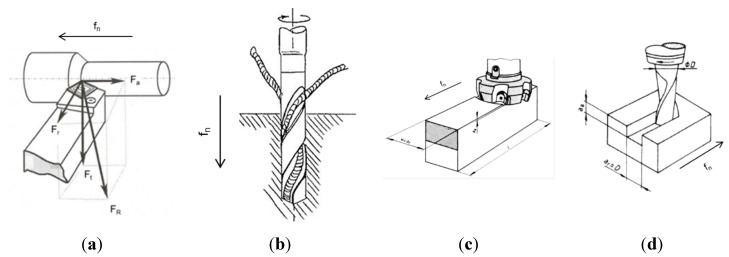
Some typical machining processes, (**a**) longitudinal turning [7]; (**b**) twist drilling [8]; (**c**) face milling [9]; (**d**) slot milling [10].

**Figure 5:**
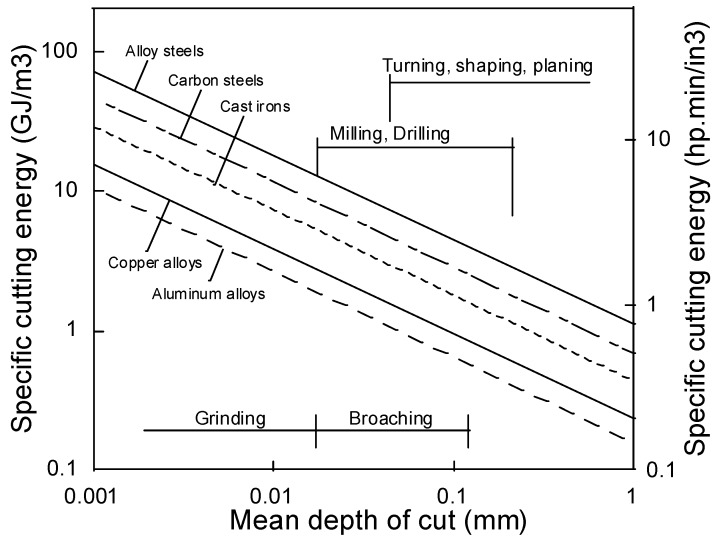
The link between energy consumption, type of machining process and metal category [4].

Many machining processes exist today, but the most frequent in traditional production may be turning and milling. Other common machining processes include drilling, grinding, broaching and shaping. The applied techniques of longitudinal turning, twist drilling, face milling and slot milling are shown in Figure 4. What is unique for turning, out of these four processes, is that the work piece rotates whilst the cutting tool only moves in the feed direction. This is in contrast to twist drilling, face milling and slot milling, where the work pieces are still and the cutting tool rotates. However, all machining processes behave different when it comes to metal fracture during machining *i.e.*, material removal. As they are different, they also consume different amounts of energy (see Figure 5). It can be observed that turning corresponds to a manufacturing process that is associated with larger depths of cuts. However, fine machining processes as grinding and broaching only enables small cutting depths. Therefore, the volumetric energy consumption is higher for the fine machining operations (grinding, broaching) than that for the rougher techniques (turning, milling, drilling, *etc.*). In addition, the energy consumption is higher for alloyed steel (e.g., stainless steel) than for e.g., aluminum alloys due to an increased mechanical strength, toughness, *etc.*, cp. Figure 5.

**Figure 6:**
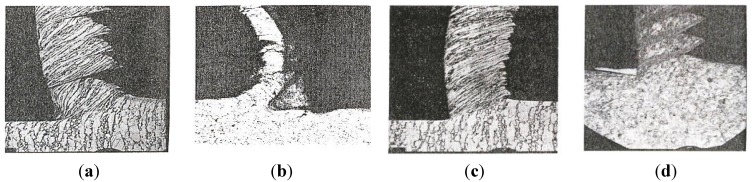
(**a**) Discontinuous chip, Cutting speed +; (**b**) continuous over a built-up edge, Cutting speed ++; (**c**) continuous chip, Cutting speed +++; and (**d**) segmented chip [13], Cutting speed ++++.

Chip formation during metal cutting can be considered as the main concept to label the mechanisms of metal fractures during machining. Chips can be produced in many shapes, but there are four overall types of chips *i.e.*, discontinuous, continuous over a built-up-edge, continuous and segmented [11,12], as shown in Figure 6.

A discontinuous chip formation occurs at lower cutting speeds. It is due to strain hardening of the work piece material, which in turn results in friction against the cutting tool. Therefore, the work piece material tends to stick. New, incoming materials at the primary shear zone interact with the remaining work piece material, which consequently bulges out from the cutting tool. Such material movements initiate a crack formation which in the end results in the formation of a chip.

A continuous chip formation over a built-up-edge arises in general at intermediate cutting speeds, induced by material adhering to the cutting tool. The built-up edge consists mainly of a strain-hardened work piece material; this is why it is strong enough to function as a “new” cutting edge, which allows a continuous chip flow.

A continuous chip formation is characterized by a uniform and stable process, due to a machining at slightly higher cutting speeds than for the built-up-edge formation. An increase of the cutting speed governs a higher machining temperature, which in turn eliminates the built-up-edge.

A segmented chip formation occurs at high cutting speeds when the deformation is localized to the compressed shear bands *i.e.*, the segments. It is believed that the chips are sheared off due to very high loads and that they are then sub-sequentially welded together. The force can be larger in this chip formation mechanism compared to the others which also explains why the chips are more irregular in shape. The chip formation is mainly influenced by the cutting speed and material properties of the work piece e.g., the hardness, ductility, work hardenability, microstructure and the characteristics of non-metallic inclusions.

**Figure 7:**
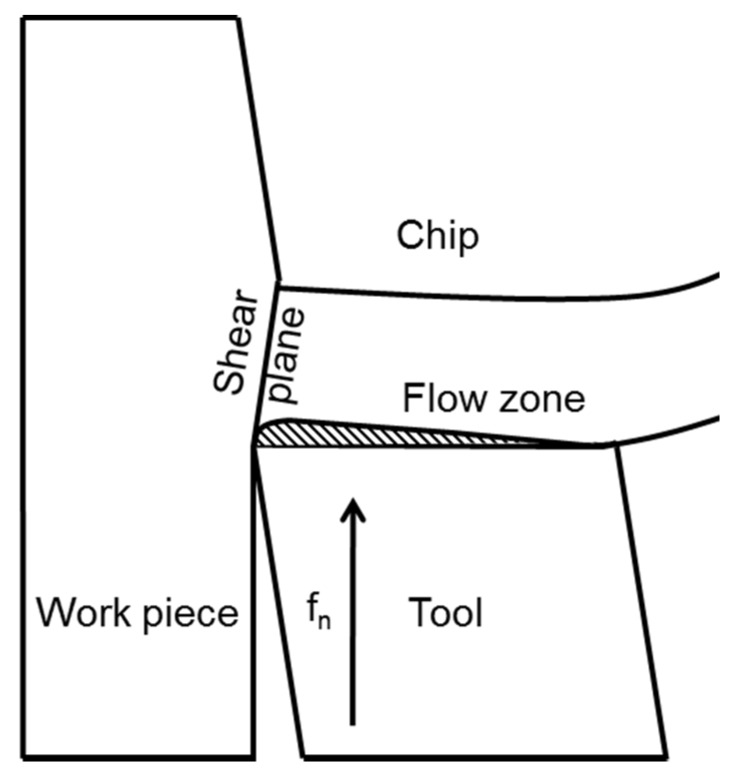
The chip formation process during turning [14].

Non-metallic inclusions play an important role as they can serve to ease the chip formation process. A lot of understanding in this case is appreciated to Kiessling [14], who explained that metal cutting can be significantly improved when the inclusions:

1. Act as stress raisers in the shear plane, which cause a crack formation. This, in turn, leads to embrittled chips that are easily broken. In addition, the length of the contact zone between the chip and the cutting tool is reduced. Thus, is advantageous for the tool wear resistance.
2. Are active in the metal flow zone (see Figure 7, where f_n_ is the feed direction) and contributes to shearing of the metal. However, an appropriate balance of inclusions is necessary to avoid an increased tool wear rate.
3. Form a diffusion barrier, isolating the rake face from diffusion induced chemical tool wear at high temperatures.
4. Act as lubricant which protects the flank face of a cutting tool from an abrasive wear.

**Figure 8:**
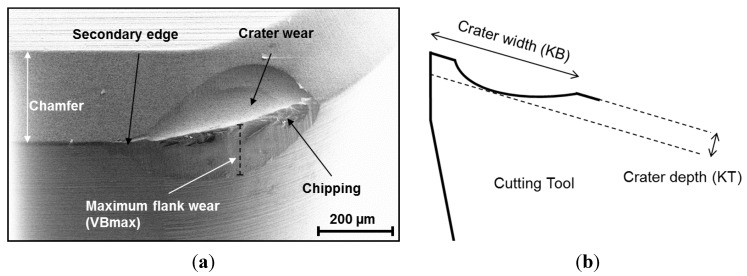
(**a**) Examples of tool wear patterns after turning of a hardened steel using a poly-crystalline boron nitride (PCBN) cutting tool; and (**b**) How to measure the crater depth (KT) and crater width (KB) [16].

Tool wear will arise during metal cutting processes due to the high forces and the elevated temperatures that arise. The wear of the cutting tool can appear in different patterns usually described as an edge chipping as well as a fracture and flank, crater and notch wear [15]. The most common wear patterns may be the flank wear (FW) and crater wear (CW). These are shown in Figure 8. The flank wear is believed to be caused by an interaction between hard and abrasive non-metallic inclusions from the work piece. Maximum flank wear (VBmax) is the usual measure of a flank wear. A crater wear is typically quantified as depth (KT) or width (KB), as shown in Figure 8. The crater wear is linked to the intense contact with the work piece material during the chip formation process.

The level of mechanical properties and machinability of different steels depends on the following characteristics of the machined steels: (i) steel composition (concentrations and distributions of different dissolved elements); (ii) microstructure of steel (grain size and microstructure); and (iii) non-metallic inclusions (NMI) in steel (composition, number, size, morphology, distribution). This review is focused on a detailed consideration of characteristics of different non-metallic inclusions in various steel grades and their link to machinability. Furthermore, this chapter considers the characteristics of different non-metallic inclusions and their behavior in the cutting zone during mechanical machining, and their effect on machinability of various steel grades.

### 2.2. Behavior of Non-Metallic Inclusions in the Cutting Zone

**Figure 9:**
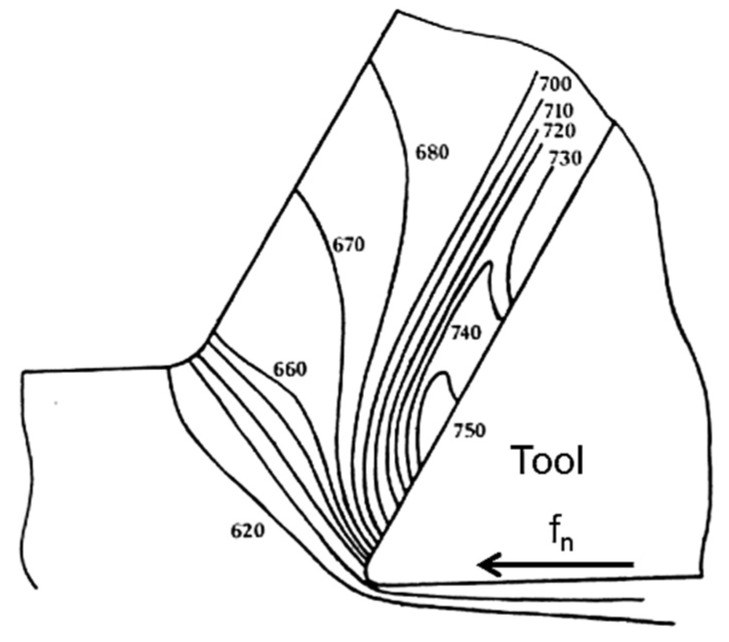
Typical temperature distribution in the cutting zone (in °C) [17].

It is well known that the temperature of the cutting tool, the chip and the work piece can be significantly increased during machining of steel and that it can reach levels typically of 750 °C [17]. The temperature level depends on several machining parameters (such as the speed of cutting, depth of cut and feed rate) and the characteristics of steel (e.g., thermal diffusivity and hardness). An example of a typical temperature distribution in the cutting zone is shown in Figure 9, where f_n_ is the feed direction.

In addition, an existence of significant temperature gradients within different zones of the work piece and the chip leads to different thermal expansions of the steel matrix and of the non-metallic inclusions. A heating that is followed by cooling within different zones of a work piece or a chip, due to mechanical machining, will induce tessellated stresses in the steel matrix near the inclusions. It can be expected that higher stresses will promote strain as well as nucleation and/or propagation of cracks in the steel matrix. Moreover, it is safe to assume that the values of these stress fields and cavities in the steel are proportional to the size and number of the present inclusions. The values of these stresses depend largely on the difference in the thermal expansion coefficients, α, between the steel matrix and the non-metallic inclusions. The additional stresses in non-metallic inclusions and the steel matrix that arises during heating and cooling are schematically shown in Table 1.

**Table 1:** Formation of additional stress fields, cavities and pores around non-metallic inclusions (NMI) and steel matrix due to different thermal expansions during heating and cooling.

Coefficient of Thermal Expansion	Group 1: α*_NMI_* *<* α*_steel_*	Group 2: α*_NMI_* ~ α*_steel_*	Group 3: α*_NMI_* > α*_steel_*
Heating	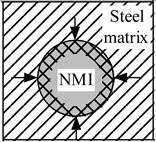	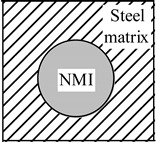	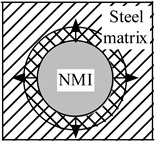
	Stress in NMI	No effect	Stress in steel matrix around NMI
Cooling	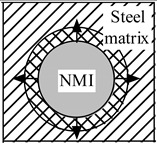	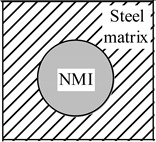	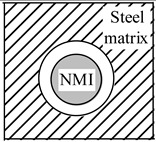
	Stress in steel matrix around NMI	No effect	Formation of cavity and pores around NMI

The non-metallic inclusions from Group 2 (α*_NMI_* ~ α*_steel_*) are expected to have no additional effect on the machining process, due to the similar thermal expansion or contraction (reduction) of the steel matrix and inclusion. However, inclusions from Group 1 (α*_NMI_* < α*_steel_*) and Group 3 (α*_NMI_* > α*_steel_*) can significantly promote a degradation of the steel matrix during heating and a subsequent cooling during machining. This is due to the fact that the inclusions can help to propagate crack lines in the cutting zone. The power consumption can thereby be reduced within metal removal processes, due to the formation of stress fields, cavities and pores in steel matrix around the inclusions.

**Figure 10:**
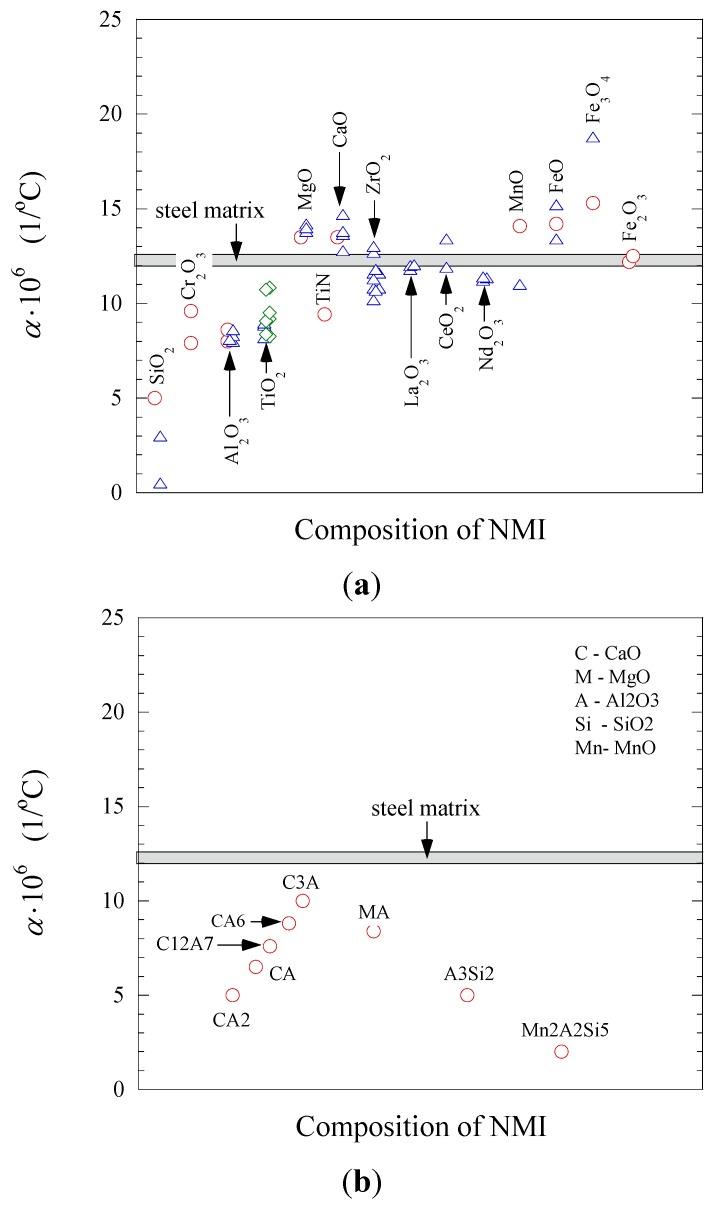
Thermal expansion coefficients, α, for some (**a**) oxides and nitrides; and (**b**) complex oxides CaO-Al_2_O_3_-MgO-SiO_2_-MnO with respect to the steel matrix. References of point marks: red circle—[18], blue triangle—[19], green diamond—[20].

**Figure 11:**
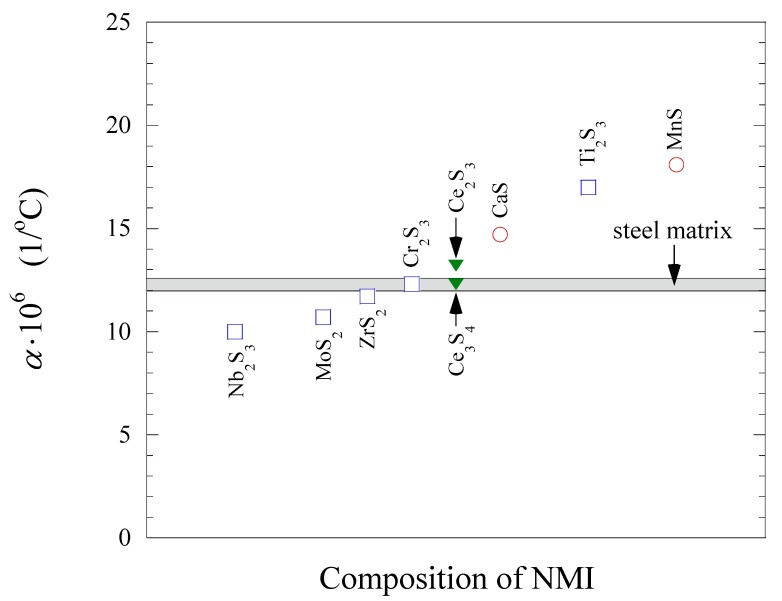
Thermal expansion coefficients, α, for some sulfides in comparison to the steel matrix. References of point marks: red circle—[18], blue square—[21], green filled triangle—[22].

The magnitude of the effect of non-metallic inclusions on the improvement of the machinability of steel matrix depends on the difference between the α*_NMI_* and the α*_steel_* values. Figure 10 and Figure 11 shows the values of the thermal expansion coefficient for a steel matrix and some oxide and sulfide non-metallic inclusions, respectively, which were reported in previous work [18,19,20,21,22]. The non-metallic inclusions that have different compositions and α coefficient than that of the steel matrix can effect on the steel machinability to some extent. However, it should be pointed out that the value of the α coefficients can vary for different steel grades, depending on the contents of carbon and alloying elements. Therefore, the influence of the same inclusions can vary depending on steel grade. For instance, the oxides and sulfides of rare-earth metal (REM) and Zr have α coefficient values of 10.7 × 10^−6^–13.4 × 10^−6^ 1/°C, which are much closer to that for low alloyed and carbon steels (10.1 × 10^−6^–11.8 × 10^−6^ 1/°C) [23] than the value for MnS (~18.1 × 10^−6^ 1/°C). Thus, the expectation is that MnS inclusions can induce tessellated stresses in these steel grades (α ~ 12 × 10^−6^ 1/°C) to a larger extent in comparison to the oxides and sulfides including REM and Zr elements. However, the thermal expansion coefficients for stainless steels (16.0 × 10^−6^–17.8 × 10^−6^ 1/°C) [23,24] and high alloyed steels (24.5 × 10^−6^–24.7 × 10^−6^ 1/°C) [24] are significantly larger compared to those of low alloyed and carbon steels. In this case, the α*_MnS_* is close to those values corresponding to stainless and high-alloyed steels. Therefore, the effect of MnS inclusions on improving of machinability will be smaller in comparison to the oxides and sulfides of REM and Zr. It can be explained by the significantly lower magnitude of the difference between the values of α*_MnS_* and α*_steel_* for the stainless and high alloyed steels.

It should be pointed out that the total effect of different inclusions on the machinability and final mechanical properties of various steel grades also depends on such characteristics of non-metallic inclusions as hardness, deformability, number, size, morphology, distribution in steel, *etc.* The comparative characteristics of main different non-metallic inclusions are summarized and listed in Table 2. It can be seen that various non-metallic inclusions in steels have very different characteristics. Therefore, for improvement of machinability of steel without significant reduction of mechanical properties, characteristics of non-metallic inclusions should be optimized for each group of steel grades. The qualitative influence of different non-metallic inclusions on some mechanical properties and mechanical machinability of steels are listed in Table 3.

**Table 2:** Comparative characteristics of different non-metallic inclusions in steels.

Operation	Si-Deoxidation	Al-Deoxidation	Ca-Treatment (Modification of Oxides)	Addition of S	Ca-Treatment (Modification of Sulfides)	REM-Addition (Modification of Sulfides)	Addition of Zr, Ti, V, Nb or B
Non-metallic inclusions (NMI)	SiO_2_, SiO_2_-MnO-...	Al_2_O_3_, Al_2_O_3_-MgO	CaO, CaO-Al_2_O_3_, CaO-Al_2_O_3_-... CaO-SiO_2_-…	MnS, (Mn,Fe)S	Ca(O,S), CaS, (Ca,Mn)S	REM-O_x_, REM(O,S)_x_, REM-S_x_	ZrN, Zr(N,C), TiN, Ti(N,C), BN, B(N,C), BC, *etc.*
Formation of NMI in steel	Partially in liquid steel. Partially during solidification of melt due to high content of soluble O (~60–80 ppm)	In liquid steel	In liquid steel	During solidification of melt (large size sulfides—mostly in final solidified zones)	In liquid steel	In liquid steel	Mostly during solidification of melt. Partially after solidification of melt
Size of NMI in cast metal	1–8 µm	0.1–8 µm	1–25 µm	0.5–30 µm	1–5 µm	1–3 µm	0.01–7 µm
Condition/ Morphology *	Liquid or solid/SP and RE	Solid/Mostly RE and IR	Liquid or solid/Mostly SP	Solid/Mostly RE and IR	Solid/Mostly SP	Solid/SP and RE/IR	Solid/Mostly RE and IR
Distribution in steel	Mostly homogeneous	Mostly homogeneous	Mostly homogeneous	Mostly in final solidified zones, S inhomogeneity	Mostly homogeneous	Mostly homogeneous	Mostly on grain boundaries
Cluster formation	No	Very easy. Size of clusters 10–1000 µm	No	Dendrite or coral shape sulfides (Type II and IV), 10–100 µm	No	REM-oxides. Size of clusters 10–300 µm	TiN-“clusters”. Size of clusters 5–30 µm
Hardness of NMI (kg/mm^2^)	Middle/Low (~1600)	High (~3000)	Middle/Low (850–1200)	Low	Middle/Low	High	High
Deformability of NMI	Low at T < 900 °C High at T > 1000 °C	No at T < 1300 °C Low at T ≥ 1500 °C	No at T < 1200 °C High at T ≥ 1300 °C	Very high at T < 1000 °C	No at T < 1200 °C Low at T ≥ 1300 °C	Very low	Very low
Thermal expansion, α (×10^−6^ 1/°C)	Very low (0.5–5.0)	Low (8.0–8.6)	Low/middle (for CaO-Al_2_O_3_ 5.0–10.0)	MnS—high (18.1)	CaS—high (~14.7)	REM-O_x_—middle (11.2–13.4) REM-S_x_—middle (12.3–13.2)	TiN—low (~9.4)

Notes: *: SP: spherical shape of inclusions, RE: regular shape of inclusions, IR: irregular shape of inclusions.

**Table 3:** Qualitative influence of different non-metallic inclusions (NMI) on some mechanical properties and mechanical machinability of steels.

Non-Metallic Inclusions (NMI)	SiO_2_, SiO_2_-MnO-...	Al_2_O_3_, Al_2_O_3_-MgO	CaO, CaO-Al_2_O_3_, CaO-Al_2_O_3_-..., CaO-SiO_2_-…	MnS, (Mn,Fe)S	Ca(O,S), CaS, (Ca,Mn)S	REM-O_x_, REM(O,S)_x_, REM-S_x_	ZrN, Zr(N,C), TiN, Ti(N,C), VN, V(N,C), BN, B(N,C), BC
Effect of NMI on the mechanical properties of steel.	No or some anisotropy of mechanical properties of steel due to low elongation of silicate inclusions during deformation.	No anisotropy of mechanical properties of steel.	- No anisotropy of mechanical properties of steel. - Increasing of ductility and toughness of steel.	- Very high (×1.5~10) anisotropy of mechanical properties of steel due to elongation of MnS during deformation. - Significant decreasing of toughness, weldability and level of cold brittleness of steel. - Large corrosion of steel.	- Low or no anisotropy of mechanical properties of steel. - Increasing of ductility and toughness of steel.	- Low or no anisotropy of mechanical properties of steel. - Improved ductility, toughness of steel, fatigue resistance of steel, impact strength, cold brittleness threshold. - Improved corrosion resistance of steel.	- No anisotropy of mechanical properties of steel. - Significant increase of strength of steel, decreasing of toughness of steel.
Effect of NMI on the machinability of steel.	- Cutting force and power consumption is very high. - Chip formation is poor or normal. - Tool wear rate is very high due to hard and abrasive NMI.	- Cutting force and power consumption is very high. - Chip formation is poor or normal. - Tool wear rate is very high due to hard and abrasive NMI.	- Cutting force and power consumption is high. - Chip formation is good or normal. - Tool wear rate is very low due to soft NMI and good lubrication effect.	- Cutting force and power consumption is low or middle. - Chip formation is good. - Tool wear rate is low due to soft and ductile NMI and some lubrication effect.	- Cutting force and power consumption is low or middle. - Chip formation is normal. - Tool wear rate is low due to some lubrication effect of NMI.	- Cutting force and power consumption is high or middle. - Chip formation is poor or normal. - Tool wear rate is low or normal due to some lubrication effect of NMI.	- Cutting force and power consumption is middle. - Chip formation is normal. - Tool wear rate is low, normal or high depending on hardness, size and number of NMI.

## 3. Non-Metallic Inclusions in Different Steels and Their Link to Machinability Tests

This part of the review was focused on an analysis of previous published studies with respect to the relationship between non-metallic inclusions and the mechanical machinability of different steels. The purpose was to gather information about how inclusion characteristics in work piece materials is interpreted using machinability tests.

This investigation was initially focused on the evaluation of non-metallic inclusions in clean steels and stainless steels and their correlation to their machinability output. However, it was soon realized that very little has been reported on for such steel grades. More information is given for so called “free-machining steels” *i.e.*, with high sulfur content. Such steels can easily be machined but have significantly lower mechanical properties and corrosion resistance. Table 4 presents an overview of published studies which considered the correlation of inclusion characteristics in different steel grades and their machinability parameters such as the tool life (TL), tool wear (TW), cutting forces (CF), chip characteristics (CC), surface roughness of work piece after machining (SR), *etc.*

According to this overview, it was found that the parameters tool wear and tool life (More specifically, about 85% and 55% of articles reported results of these machinability tests) are the most common for evaluation of the effect of non-metallic inclusions on machinability of various steels. Parameters such as cutting forces and chip characteristics during mechanical machining are reported in about 35% of the investigated articles. Moreover, to evaluate the surface roughness of a machined work piece or to measure the cutting temperature seems to be a less common practice.

It can also be seen that most of the published studies are focused on an investigation of the influence of different non-metallic inclusions on the machinability of free-machining steels and “quench and tempering” (Q & T) steels (e.g., SS 2541 steel, *etc.*). Fewer articles report results for clean steels and duplex stainless steels. Moreover, the typical non-metallic inclusions, which characteristics are investigated and compared in the most studies regarding to machinability of steels include MnS, (Mn,Ca)S, Al_2_O_3_, (CaO)-(Al_2_O_3_), CaO-Al_2_O_3_-SiO_2_, REM-O, REM-S, and their various combinations. The discussions in the following sections are mainly based on the findings presented in the articles given in Table 4.

**Table 4:** Overview of published studies which considered the correlation of inclusion characteristics in different steel grades and their machinability parameters.

Ref.	Year	Steel Grade ^a^	Inclusion Characteristics	Machinability Parameter ^b^	Main Result
[25]	1995	“Clean”, carbon	(Mn,Ca)S, elongated, (CaO-Al_2_O_3_), globular	TL	Ca-treatment improves machinability
[26]	1995	“Clean”, carbon, M-steel	(Mn,Ca)S, elongated, (CaOAl_2_O_3_), globular	TL, TW	Ca-treatment improves machinability
[27]	1981	Ca-treated, carbon, M-steel	CaO-Al_2_O_3_, globular, CaO-Al_2_O_3_-SiO_2_, anorthite, globular	TL, TW	Ca-treatment improves machinability
[5]	2007	Ca-treated, medium carbon steel, 0.35%–0.40% C, 0.02%–0.04% S	Al_2_O_3_-MgO, regular, CaO-Al_2_O_3_, 12CaO-7Al_2_O_3_, globular	TW	Ca-treatment improves machinability
[28]	1993	SS 2541, Q & T	MnS, elongated, (Mn,Ca)S, globular, (CaO-Al_2_O_3_)-(Mn,Ca)S and CaO-Al_2_O_3_-SiO_2_, globular	TL, TW	Decreased flank wear progression due to Ca-treatment
[29]	2013	42CrMo, Q&T, 0.42% C, 0.0067% S	BN, globular, 5–20 µm	TW, CC	BN improved the machinability (drilling)
[30]	1999	AISI 4140, Q&T, 0.0017%–0.0030% Ca, 0.4% C	MnS, (Ca,Mn)S, globular	TL, CF	Reduced torque and adhesion due to Ca-treatment
[1]	1993	SS2541, ~0.35% C, 0.035% S 825B BB, 1% C, 0.011% S	MnS, (Ca,Mn)S, (CaO-Al_2_O_3_)-MnS, AlCaMnS	TW, CF	The protective (Mn,Ca)S layer reduced the crater wear
[31]	1984	SS 2506, CH, S, Ca ~0.2% C, 0.04%–0.09% S, 0.0003%–0.0054% Ca	MnS, elongated, (Mn,Ca)S~elongated, (CaO-Al_2_O_3_)-(Mn,Ca)S and (CaO-Al_2_O_3_-SiO_2_)-(Mn,Ca)S, globular	TL, TW	S and Ca-treatment improves machinability
[32]	1986	SS 2506, CH, Ca additions 0.04%–0.09% S	MnS, elongated, (Ca,Mn)S, (CaO-Al_2_O_3_)-(Mn,Ca)S, globular	TL, TW	Ca-treatment improves machinability
[33]	2001	40 CrMnMo8 Carbon 0.4% C, 0.008%–0.067% S	MnS, elongated, 20–100 µm, oxides, globular, 10 µm	TL, TW, CC	S addition increased the machinability by 40%
[34]	2001	AISI 4340 ~0.4% C, 0.012%–0.034% S, 0–50 ppm O, 0–25 ppm Ca	(CaO-Al_2_O_3_)-(Mn,Ca)S, globular, 2–10 µm	TW, CF, CC	Ca-treatment indicates ridge formation after hard part turning
[35]	1984	Structural steel	S, Se, Pb, Ca	TL	Additions of S, Se, Pb, Ca improved the machinability
[36]	1975	Free mach, 0.3% S	MnS, elongated	TL, TW, CF	S additions improved the machinability
[37]	1975	Free mach., 0.1% S	MnS, elongated, Al_2_O_3_, globular	TL, TW	S additions improve machinability
[38]	2006	Free mach., 0.6% C, 0.3% S	MnS, elongated, 5–40 µm MnFe(Al,Si)S	CF, CC, SR	Cold deformation may improve machinability
[39]	2012	Free mach., ~0.08% C, ~0.4% S	MnS, elongated, 10–20 µm (MnO-Al_2_O_3_)-MnS, globular, 15 µm (MnO-SiO_2_)-MnS, elongated, 20 µm	TW, CC, SR	Increased oxygen content improved the machinability
[40]	1997	Free mach., 0.4% C, 0.1% S	(Mn,Ca)S, MnS, elongated, <10 µm, (RE,Ca)_2_S_3_-(Mn,Ca)S, Re_2_S_3_-MnS, globular, <10 µm	TW	Ca and RE additions increased the machinability of free-cutting steels
[41]	1996	Free mach., stainless steel, 0.04%–0.08% C, <0.1% S, <0.01% Ca	CaO-Al_2_O_3_-SiO_2_-MnS, MnS, Gehlenite, Anorthite	TL, TW, CF	Ca and S additions increased the machinability of stainless steel
[42]	1990	Stainless steel, 316 L 0.020%–0.027% C, 0.022%–0.025% S. 0.0002%–0.0045% Ca	MnS, (Mn,Ca)S, Gehlenite: Ca_2_Al[AlSiO_7_] + MnS Anorthite + MnS, elongated phases	TW, CF, CC	Anorthite inclusions are favorable for machining of 316L stainless steel
[3]	2010	Super-duplex stainless steel, 0.017%–0.021% C, 0.005%–0.034% S. REM additions	REM-O, Oxy-sulfides, (Mn,Cr)S, globular, 2–10 µm	TL, TW	S and REM additions increased the tool life but the corrosion resistance was decreased
[43]	2011	Austenitic stainess steel, 0.10%–0.11% C, 0.02%–0.11% S. Cu, Bi, Ti additions	MnS, Ti4C2S2, CuO, Bi, globular	TW, CF, CC	S, Bi, Cu and Ti additives improved the machinability

Notes: ^a^: steel grades: M-steel: Machinability improved steel; Q & T: Quench and tempered; CH: Case hardened steel; BB: Ball-bearing steel; ^b^: machinability parameters such as the tool life (TL), tool wear (TW), cutting forces (CF), chip characteristics (CC), and surface roughness of work piece after machining (SR).

## 4. Control and Correction of Non-Metallic Inclusions for Improving the Machinability of Steel

Today, there are many techniques available in the steelmaking industry for a correction and control of the characteristics of non-metallic inclusions. Based on an overview of present publications, it can be concluded that the main techniques involve: (1) an increase of the S content in the steel for a larger amount of sulfide inclusions; (2) a modification of sulfide inclusions (MnS) by a treatment of liquid steel with Ca, REM or Zr; (3) a modification of present oxides in the liquid steel (such as Al_2_O_3_, Al_2_O_3_-MgO, SiO_2_, *etc.*) by Ca-treatment; and (4) an addition of other elements (such as Se, Te, B, *etc.*) for a specific objective. Some of these techniques are discussed below.

### 4.1. Increasing the S Content of Steel

Improving the machinability by sulfur additions is a traditional approach. Sulfur is added in the range of 0.08–0.13 wt.% (occasionally till 0.33 wt.%) to several steels for machinability improvement, as follows from Table 5 [44]. An addition of S improves the machinability of steels due to the formation of an additional number of sulfides e.g., MnS which are precipitated mostly during solidification of liquid steel. Therefore, it is also essential to consider the content of manganese in the steel.

**Table 5:** Content of C, S and Mn in some common steel grades (wt.%) [44].

AISI Steel Grade	C	S	Mn
1010	0.07–0.14	0.05 (max)	0.25–0.60
1110	0.08–0.13	0.08–0.13	1.00–1.30
1037	0.31–0.38	0.05 (max)	0.70–1.00
1137	0.32–0.39	0.08–0.13	1.35–1.65
1045	0.42–0.50	0.05 (max)	0.60–0.90
1144	0.40–0.48	0.24–0.33	1.35–1.65

**Figure 12:**
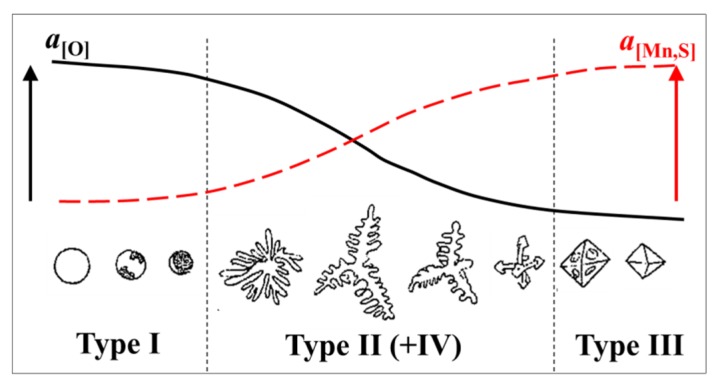
Schematic illustration of the relationship between activities of O, Mn and S and the morphology of oxy-sulfides and sulfides in steel [47,48].

In the as cast condition, MnS inclusions can be classified into three main morphologies [45,46] (see Figure 12):

Type I: globular, when the oxygen solubility is high and the sulfur solubility is relatively low. Such inclusions are formed by a monotectic reaction in rimmed and semi-killed steels (when aluminum in the steel is less than 0.001 wt.%).
Type II: formed in the interdendritic spaces of austenite with a fan-like morphology. In addition, most commonly formed at grain boundaries of steel. These are formed in aluminum killed steels, without an excess amount of aluminum, as the aluminum content is about 0.007% in the steel.
Type III: angular inclusions are formed as isolated particles in the interdendritic spaces, when excess aluminum is used for deoxidation resulting in about 0.038 wt.% aluminum in the steel.

**Figure 13:**
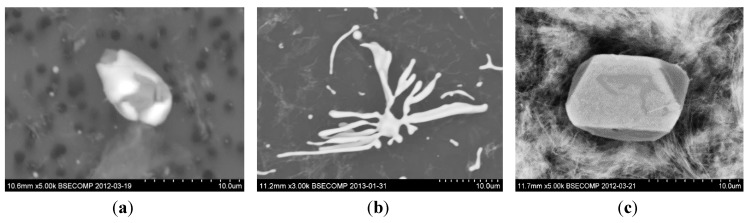
Typical morphology of different sulfides. (**a**) Type I oxy-sulfide; (**b**) Type II (+IV) eutectic MnS; (**c**) Type III regular MnS [49,50].

Moreover, in some studies [47,48], Type IV sulfides having dendritic or skeleton shapes were discussed. Typical photographs of different sulfides in steel samples are shown in Figure 13 [49,50].

**Figure 14:**
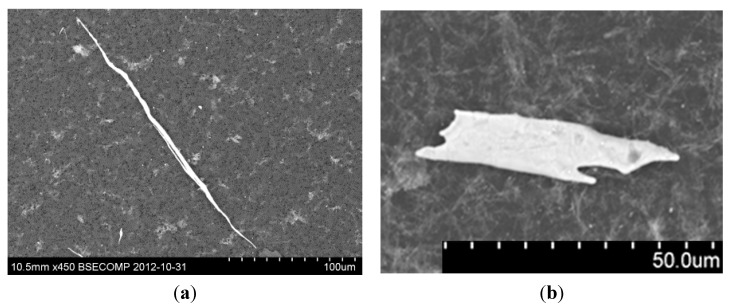
The typical morphology of a deformed sulfide [49]. (**a**) Rod-like and elongated MnS; and (**b**) Leaf-like deformed MnS inclusion.

Type I sulfide inclusions contain usually an oxide core and are therefore harder than Type II sulfides. Type I oxy-sulfides are usually present in steel as individual particles while Type II (and Type IV) MnS inclusions are formed by an eutectic reaction in the interdendritic spaces. In addition, Type II sulfides can deform to a larger extent than the inclusions of Type I during hot working of the steel. Therefore, they may be more harmful to the materials mechanical properties. Thus, MnS inclusions of Type II and III become elongated during rolling or other deformations of steel. These elongated inclusions introduce an anisotropy of the mechanical properties of steel which leads to an inferior strength, ductility and toughness in the short transverse direction. A typical shape of deformed MnS inclusions after rolling is shown in Figure 14 [49].

**Figure 15:**
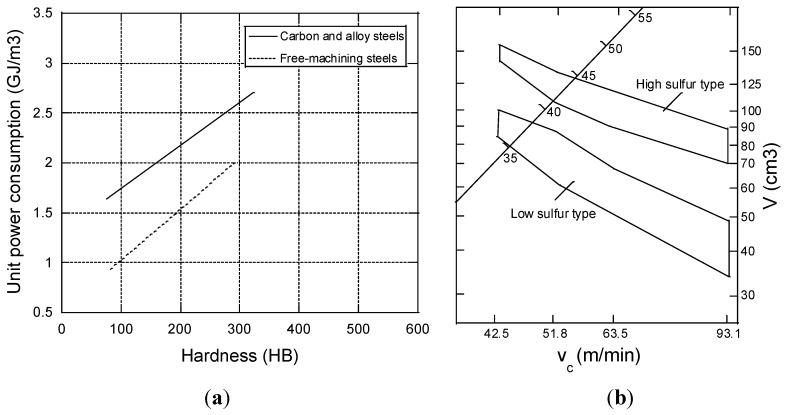
(**a**) Effect of sulfur content and hardness on the unit power consumption for different steel grades [44]; (**b**) Influence of sulfur content (high sulfur ~ 0.1%–0.3% S and low sulfur < 0.05% S) on the machinability in milling of case hardening steels [51].

Sulfides that precipitate in steel have a lower shear strength in the cutting zone in comparison to the steel matrix. In addition, sulfide inclusions form stress fields in the steel matrix that weaken the steel. It can lead to a high rate of deformation within local zones of the steel matrix during machining. Moreover, MnS inclusions have a positive influence on the machinability, as they are soft in comparison to the steel matrix and act as voids. Hence, they separate from the surrounding steel matrix when the chip is formed by the action of the cutting tool. As a result, the chips are shorter in length which promotes an easier removal. There exists a lower friction between the tool and chip, which decreases the total power consumption during machining. Since the chips are removed at shorter lengths with lower friction coefficient, the surface finish of the component will also be superior for the sulfur containing steels compared to the other steels. As a result, the removed volume of metal (V) at the given cutting speeds (v_c_) of the high sulfur steels is drastically higher (at about 40%) in comparison to other steel grades, as shown in Figure 15 [44].

A sulfur addition promotes a formation of soft MnS inclusions. An increased sulfide level in steel corresponds to an increased tool life, as is well-known. One example is given in reference [3] where three steels of type 25Cr/7Ni stainless steels were compared. Their sulfur contents were 0.005%, 0.0340% and 0.1181%, respectively. This increase in sulfur content resulted in an increased volume fraction of inclusions by 25%–75%. Therefore, the tool life in single point turning increased correspondingly with 2–12 times in comparison to that the low-sulfur steel. However, the critical pitting temperature decreased from 80 to 60 °C for the high-sulfur steel grade. It means that the improved machinability was gained in favor to the corrosion resistance.

In another case, the machinability of a free-cutting stainless steel was compared with an ordinary steel grade [41]. It was found that due to an increase of S from 0.04% to 0.1%, the flank wear was reduced with about 50%, after 30 min of longitudinal turning. The main cutting force (F_z_) for each grade was also compared. It was 25% lower for the free-cutting steel than that of the ordinary grade. Such a large difference in S content is expected to generate the observed differences in machinability. Thus, reported results are expected.

### 4.2. Modification of Sulfide Inclusions by Addition of Ca, REM or Zr

**Figure 16:**
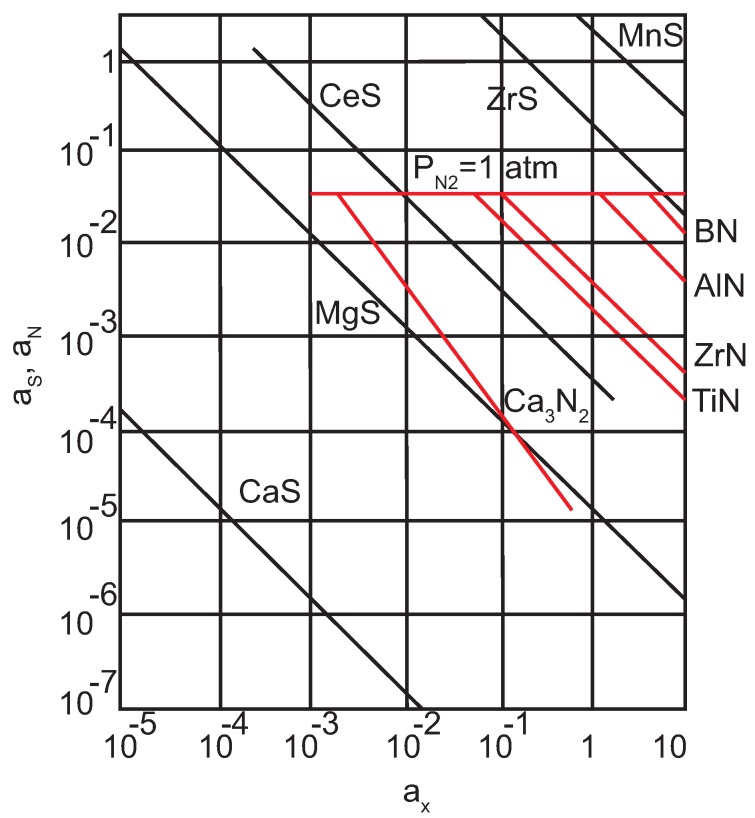
Activity of and N in equilibrium with various elements in liquid Fe at 1600 °C [56].

The Deformation of MnS inclusions in steels increases the interphase surface between inclusion and the steel matrix. This can lead to significantly decreased performance properties of steel e.g., plasticity and toughness. Moreover, MnS inclusions are associated with pitting corrosion of commercial stainless steels and can act as initiation sites [52,53,54,55]. This harmful effect of MnS inclusions on the final mechanical properties can be reduced if the sulfur content can be decreased in cast steel (with a followed decrease of the steel machinability) or by a modification of MnS inclusions by an addition of Ca, REM (Rare-Earth-Metals) or Zr in the melt. These modifying elements form more stable sulfides than MnS inclusions. In addition, as they precipitate in liquid steel, they do not deform during deformation processes. As can be seen in Figure 16, the stability of sulfides increases in the following order: MnS, ZrS, CeS, MgS and CaS.

The main purposes of the modification process of MnS inclusions are usually to:

- change the composition and properties (physical and chemical) of sulfides;
- change the sulfide morphology (globalization);
- decrease the size of the modified sulfides;
- obtain a homogeneous distribution of precipitated sulfides in the solidified steel.

The globalization and size reduction of the sulfides can lead to a reduced S-segregation and thereby the presence of no large sized MnS inclusions. Large size MnS inclusions are harmful, since they promote an anisotropy of the mechanical properties of steel. This is due to significantly elongated MnS after deformation.

#### 4.2.1. Calcium Treatment

**Figure 17:**
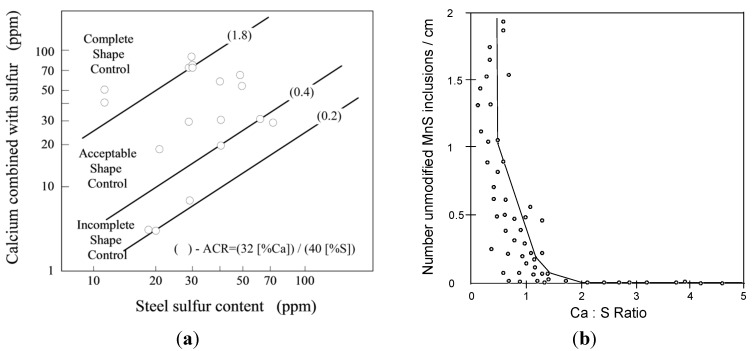
(**a**) Shape control of sulfides due to calcium addition in HSLA steel [58]; (**b**) Ca/S ratio given in wt.% correlated to the number of unmodified MnS inclusions/cm in low sulfur carbon steel [59].

Ca-treatment of different steel grades as a means to modify sulfides in the liquid steel before casting is by now considered as a well-established procedure. It should be pointed out that CaS and MnS are completely soluble with each other at the temperatures of liquid steels [57]. It enables a formation of (Ca,Mn)S inclusions in the liquid steel during Ca-treatment. Therefore, a modification of MnS inclusions may depend largely on the Ca/Mn and Ca/S ratios in the steels. Figure 17a shows the effect of the Ca/S ratio on the modification of the presented sulfides during a Ca-addition in a high strength low alloyed (HSLA) steel and a low sulfur carbon steel [58,59]. Although the data points are very scattered, it is apparent that the atomic concentration ratio (ACR = (32·[wt.% Ca])/(40·[wt.% S])) in Figure 17a) more than 1.8 provides a complete sulfide shape control. The ACR value in the range from 0.4 to 1.8 gives an acceptable shape control of sulfides in the steel. It can be seen in Figure 17b) that the number of unmodified MnS inclusions is negligible small in the low sulfur carbon steel with ratio of (wt.% Ca)/(wt.% S) > 1.44 (which corresponds to ACR > 1.8). However, if the (wt.% Ca)/(wt.% S) ratio is smaller than 0.32 (ACR < 0.4), the number of unmodified MnS inclusions in steel increases dramatically. It should also be pointed out that the optimum value of the Ca/S ratio in various steel grades can be considerable different depending on the oxygen contents. This fact may be one major reason of the large scatter of the experimental results obtained in different studies.

Although calcium can be introduced as pure Ca in the liquid steel during ladle treatment, the usual practice involves additions of CaSi powder, CaSiBa powder, CaSi wire, *etc.* However, calcium has a low melting temperature (T_m_ = 810 °C), a very low solubility in liquid steel (320 ppm at 1600 °C) and a high vapor pressure (1.87 atm at 1600 °C) [60]. In addition, the standard free energies of CaO and CaS formation are both highly negative. It means that calcium has a high affinity to oxygen and sulfur in the melt. Therefore, the added Ca can be used as both a deoxidizer and a desulfurizer in the liquid steel. The competing mechanisms for formation of CaO and CaS in the melt have been studied before, from a thermodynamic perspective [25]. The following reactions were considered:

(1) $$3\left[\text{Ca}\right]\;+\;{\left({\text{Al}}_{2}{\text{O}}_{3}\right)}_{\text{inc}}\;=\;2\left[\text{Al}\right]\;+\;3{\left(\text{CaO}\right)}_{\text{inc}}$$

(2) $$3{\left(\text{CaO}\right)}_{\text{inc}}\;+\;2[\text{Al}]\;+\;3[\text{S}]\;=\;3{\left(\text{CaS}\right)}_{\text{inc}}\;+\;{\left({\text{Al}}_{2}{\text{O}}_{3}\right)}_{\text{inc}}$$

The effect of different Al contents (0.01%~0.05%) and S (0.01%~0.10%) on the equilibrium composition of inclusions was investigated in Al-deoxidized and Ca-treated carbon steel (0.4% C, 0.3% Si and 0.002% of total oxygen content) at 1600 °C [25]. It was reported that the weight fraction of CaO inclusions decreased significantly with as the content of Al and S increased. Therefore, modification of MnS during Ca-treatment of the liquid steel should be considered together with the modification of oxide inclusions e.g., Al_2_O_3_ and SiO_2_. Low sulfur steels contain S in levels somewhere between 10 ppm and 50 ppm. In addition, CaS are primarily formed due to its stronger affinity to sulfur than to Mn in this case. However, a minor amount of MnS is also formed. An increased sulfur content of e.g., 300 ppm alters the inclusion balance. It becomes impossible to only bind sulfur solely in CaS. Instead, many MnS inclusions are formed though often combined with CaS which results in the formation of (Mn,Ca)S. (Mn,Ca)S inclusions are less ductile compared to MnS. This is due to the calcium content. In addition, these are more globular in shape after casting and rolling. Similar results were reported in another study [61]. It was found that a Ca-treatment of steels that contain sulfur provides the formation of (Mn,Ca)S inclusions. The (Mn,Ca)S were less elongated during deformation of steel in comparison to pure MnS, *i.e.*, Ca makes the sulfides harder than pure MnS.

However, it should be noted that the industrial application of Ca-treatment of liquid steels for modification of non-metallic inclusions are often limited by the low and unstable yield of the added Ca. This is due to the high vaporization and low solubility of Ca in the liquid steel. Therefore, some other elements such as REM and Zr having the higher vaporization temperature in the melt (for instance the boiling temperatures of some pure REM elements is varied in the range from 3130 to 3450 °C) are increasingly being used in steelmaking companies for modification of sulfide inclusions in different steel grades.

#### 4.2.2. Rare-Earth-Metals (REM) Treatment

Recently, the interest in the application of Rare-Earth-Metals (REM) for modification of NMI in different steel grades has increased sharply. A large number of experimental works (including laboratory experiments and industrial trials) have been carried out by different researchers. Here, a REM element is often described to have a high affinity to harmful impurities in steel such as O, S and N. Therefore, the influence of REM on the final properties of steel products corresponds to a reduction of the soluble contents of these harmful impurities in the steel. This is due to the formation of non-metallic inclusions with required characteristics.

Even though the melting temperature of REM elements is comparably low (798–1016 °C), the melting temperature of the formed non-metallic inclusions varies in the ranges from 1690 to ~2291 °C for oxides, from 1940 to 1990 °C for oxy-sulfides, and from 1795 to 2450 °C for sulfides [48]. When REM (lanthanum, cerium, praseodymium, neodymium, *etc.*) are added into the melt as a mischmetal or other REM-alloys, the oxides, oxy-sulfides and sulfides of the REM elements are formed as solid particles in the liquid steel. It can therefore be expected that most of the sulfur will react with REM in the melt. The precipitated REM oxy-sulfides and sulfides are small sized (about 0.5–3.0 µm) and more homogeneously distributed in the solidified steel compared to large size MnS inclusions (Type II and Type III). The latter precipitate in the last parts of a solidified steel.

The formation of non-metallic inclusions rich in REM can be described by the following reactions:

2REM + 3O ↔ REM_2_O_3_ (3)

2REM + 3S ↔ REM_2_S_3_ (4)

2REM + 2O + 3S ↔ REM_2_O_2_S_3_ (5)

REM_2_O_2_S + O ↔ REM_2_O_3_ + S (6)

REM_2_S_3_ + 2O ↔ REM_2_O_2_S + 2S (7)

**Figure 18:**
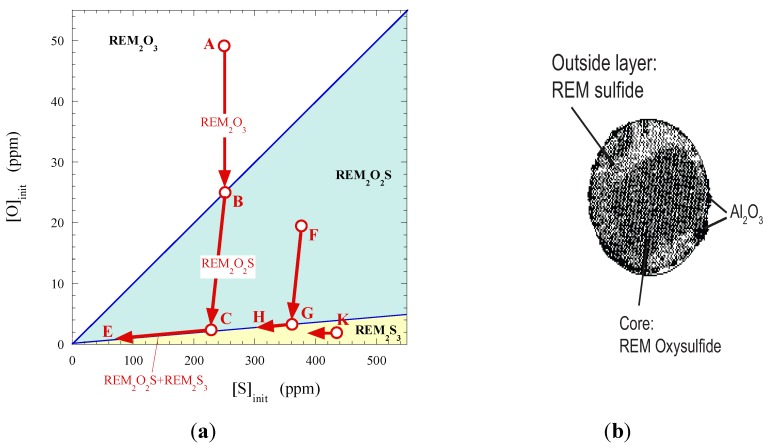
(**a**) Sequence of formation of different REM inclusions in steel after REM additions in relation to the initial contents of O and S [62]; (**b**) Typical complex REM inclusion in steel with 0.02% REM [63].

It should be noted that these reactions can occur sequentially or in parallel, depending on the local concentrations of O, S and REM in the melt. The sequence of formation of different non-metallic inclusions in steel after REM additions depends on the initial contents of O ([O]_init_) and S ([S]_init_) in the melt, as is shown in Figure 18a [62].

Only REM_2_O_3_ will precipitate in the melt if the ratio of [S]_init_/[O]_init_ is smaller than 10 (point A). During precipitation of REM_2_O_3_ oxides, the content of oxygen is reduced without a change of the S content to the point B. The REM_2_O_2_S (REMO_2_·REMS) oxy-sulfides will precipitate in the melt between the points B and C. The concentrations of O and S will decrease in the melt according to the stoichiometric ratio of O and S in the precipitated oxy-sulfide. Then, the REM oxy-sulfides and sulfides will precipitate together simultaneously and the corresponding concentrations of O and S will change according to the line CE. If the [S]_init_/[O]_init_ ratio is in the range from 10 to 100 (point F), the REM oxy-sulfides (FG segment) and oxy-sulfides + sulfides (GH line) will precipitate in the melt. If the [S]_init_/[O]_init_ ratio is larger than 100 (point K), the REM sulfides will precipitate to a cross section with the 1/100 line without changing of the O content in the melt with a following simultaneous precipitation of oxy-sulfides and sulfides. The final contents of the dissolved O and S, [O]_final_ and [S]_final_, depend on the content of added REM. As a result, the non-metallic inclusions in the steel after REM addition can have a complex (multiphase or multilayer) structure (Figure 18b): oxide or oxy-sulfide core covered by one or several layers of oxy-sulfides and sulfides. According to previous work [48], the composition of sulfide phase can change from REM_2_S_3_ to REM_3_S_4_ and further to REMS, as the sulfur content in steel decrease. Sulfides of type REM_3_S_4_ and REMS predominate in the most industrial steel grades. However, if the rest content of soluble REM in the liquid steel decreases below some critical value due, to the reoxidation or reaction with refractories and slag, some amount of S can be recovered from REM sulfides and oxy-sulfides (see Reactions (6) and (7)). In this case, the MnS inclusions can precipitate during solidification of the steel melt.

A thermodynamic evaluation regarding the formation of different REM inclusions in the liquid steel is limited because of the lack of reliable data. For instance, the values of REM activity calculated based on thermodynamic data given by different authors for a reaction of REM and S may vary 10–1000 times [48]. This big difference can be explained by the various conditions of experiments and by some other reasons.

**Figure 19:**
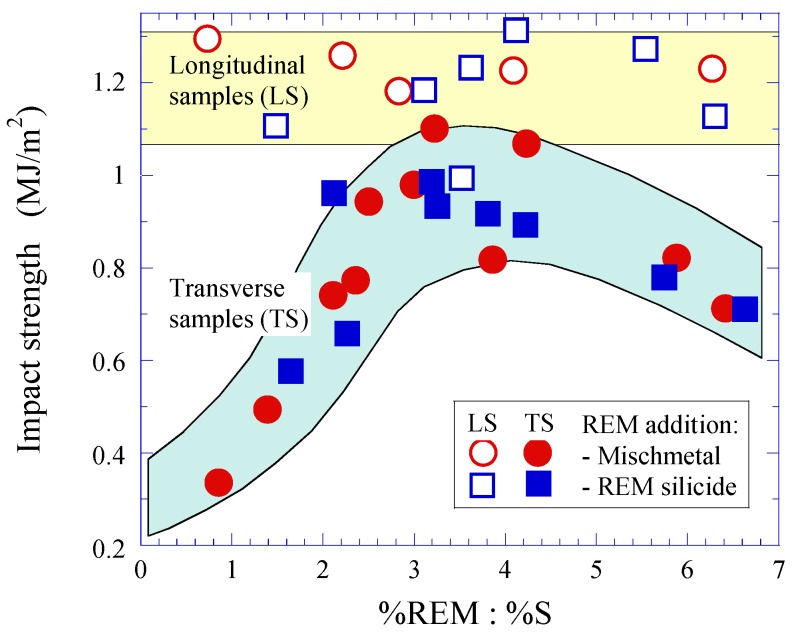
Impact strength in longitudinal (LS) and transverse (TS) samples as a function of the ratio of REM and S contents (in mass-%) in steel with additions of mischmetal or REM silicide [63].

The effect of REM additions on the mechanical properties of different steel grades are reported in many publications. For instance, it was reported in reference [63] that the impact strength in transverse samples can be increased two and more times at a ratio of added REM and S contents of about 3 (%REM:%S = 3–4 or %Ce:%S = 1.5–1.7), at which the formation of MnS inclusions was avoided. Figure 19 shows values of the impact strength in longitudinal (LS) and transverse (TS) samples as a function of the ratio of REM and S contents (in mass-%) in steel with additions of mischmetal or REM silicide [63].

According to results obtained by Ha *et al.* [55], an addition of mischmetal up to a value of 0.067% REM (at %REM/%S = 3.7) in 25% duplex stainless steel with sulfur contents of 0.016%–0.028% leads to a significant decrease of the size, area fraction and number of oxy-sulfide inclusions per unit area in steel. As a result, a resistance to pitting corrosion increased by ~34% at a REM content of 0.067%. However, further additions of mischmetal up to 0.078% REM (at %REM/%S = 4.9) decreased the resistance to pitting corrosion. This was caused by a significantly increased number and area fraction of oxy-sulfides and a change of the inclusion shape from an angular or granular shape to a needle-like shape. Wang *et al.* [64] also reported that the addition of an appropriate amount of REM alloys (0.014%–0.081% REM) in various advanced low-alloyed steels (14 MnNb, X60, 10 MnV, *etc.*) with a 0.008% S content for modification of inclusions, resulted in a deep purification and refinement of the grain size. This led an increased strength and toughness of these steel grades. Moreover, the corrosion resistance of weather resisting steels is also improved. For instance, the corrosion rate decreased on average by 17%–54% when adding 0.029% REM (%REM/%S ~ 2.0–3.6).

Among the many beneficial effects of adding REM alloys to steels, significant improvements in ductility, transverse impact strength, susceptibility to lamellar tearing in welding and bend formability have been reported [65]. Kang and Gow [66,67] have also reported an enhanced impact strength in REM treated rail steels. Moreover, they found that the REM treatment has made a significant improvement of the fatigue strength of the axle steels (0.02% S). In addition, an effort has been made to understand this improvement through the shape control of sulfide inclusions in steels. It was found that small REM inclusions were less active in both crack initiation and propagation of the fatigue fracture, compared to large MnS inclusions.

However, published experimental data obtained from laboratory experiments and industrial trials are very scattered and often contradictory. It can be explained by the imperfect technique of a REM addition, the variation of yields of an added REM and by insufficient control of the concentrations of REM, Al, O and S in liquid steel. Up to now, the optimal amount of added REM has been determined experimentally for various steel grades in different companies. Furthermore, for a given equipment and technology of steelmaking.

**Figure 20:**
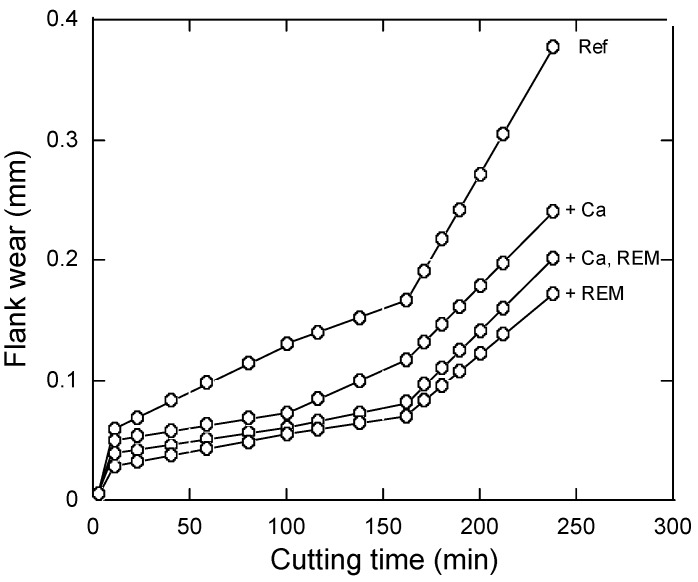
The effect of Ca and REM addition on the flank wear of free-cutting steels [40].

An improved machinability of the re-sulfurized free-cutting steels by modification of non-metallic inclusions due to addition of REM has been reported in previous work [40]. In that work, 0.027% to 0.050% REM was added with and without similar levels of Ca. It can be observed in Figure 20 that the flank wear can be decreased by an average of 32%–34% for trials with Ca-addition, by 41%–43% for trials with Ca and REM additions, and by 49%–54% for trials with REM-addition in comparison to the reference steel (without Ca and REM additions). It was found that the flank wear decreased significantly with increased REM contents in the steel. From another side, the tensile strength of the experimental steels was reduced by only 1%.

### 4.3. Modification of Oxide Inclusions by Addition of Ca

Another direction for an improvement of mechanical machinability of steels is Ca-treatment for modification of oxide-based non-metallic inclusions (such as SiO_2_, Al_2_O_3_, Al_2_O_3_-MgO, *etc.*). The Ca-treatment can improve the characteristics of the formed calcium-based oxide inclusions (e.g., composition, morphology, size, and physical and chemical properties) as well as the lubrication effect between the cutting tool and steel piece. In this case, the main advantages of a Ca-treatment for oxide-based inclusions in the liquid steel can be summarized as follows:

(i) to form the globular CaO-SiO_2_-... or CaO-Al_2_O_3_-... inclusions;
(ii) to avoid the presence of SiO_2_ oxides, which have a high deformability at T > 1000 °C and which can increase the anisotropy of mechanical properties of steel after deformation;
(iii) to avoid a formation of Al_2_O_3_ and Al_2_O_3_-MgO clusters in the liquid steel and clogging problems during casting;
(iv) an application of relatively soft CaO-SiO_2_-… and CaO-Al_2_O_3_-… inclusions as natural lubricants for cutting tools during mechanical machining for improvements of the surface quality of machined steels and to increase the tool life (reducing the tool wear *etc.*).

**Figure 21:**
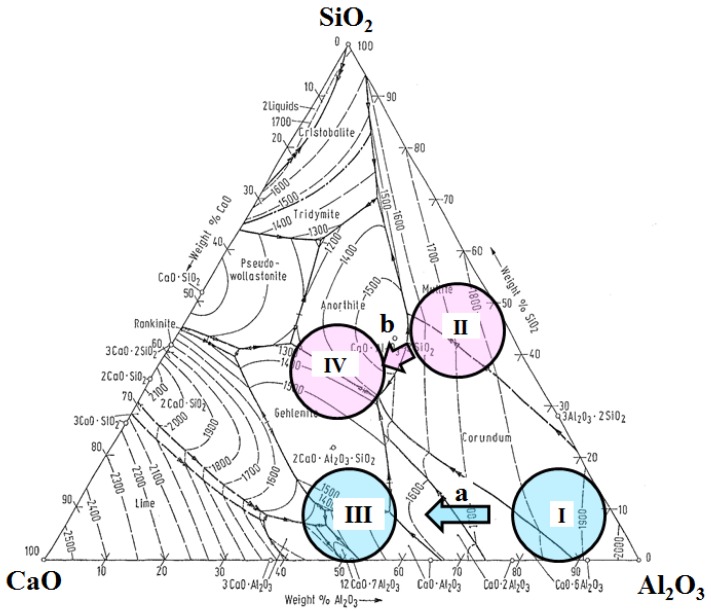
Compositions of different oxide inclusions precipitated in aluminum (**a**) and silicon (**b**) deoxidized steel grades [68].

Aluminum and silicon deoxidized steel grades have compositions of oxide inclusions within Zone I and Zone II of the ternary phase diagram, as is shown in Figure 21 [68]. Hard inclusions such as Al_2_O_3_, SiO_2_ and 3Al_2_O_3_·SiO_2_ will fracture during rolling and form hard fragments. This is detrimental for final mechanical properties and for the cutting tools during machining of such steel grades. In addition, Al_2_O_3_-based inclusions often cause nozzle clogging during casting. Calcium addition results in inclusion compositions moving in the direction of the arrows, towards Zones III and IV, respectively. Inclusions of Zones III and IV are softer and have lower melting temperatures (1400–1500 °C), a spherical shape, and better machinability properties. Thus, calcium aluminates form instead of Al_2_O_3_ inclusions in Al-deoxidized steels. In Si-deoxidized steels, mullite (Al_6_Si_2_O_13_) transforms into gehlenite (Ca_2_Al[AlSiO_7_]) or anorthite (CaA_l2_Si_2_O_8_).

**Figure 22:**
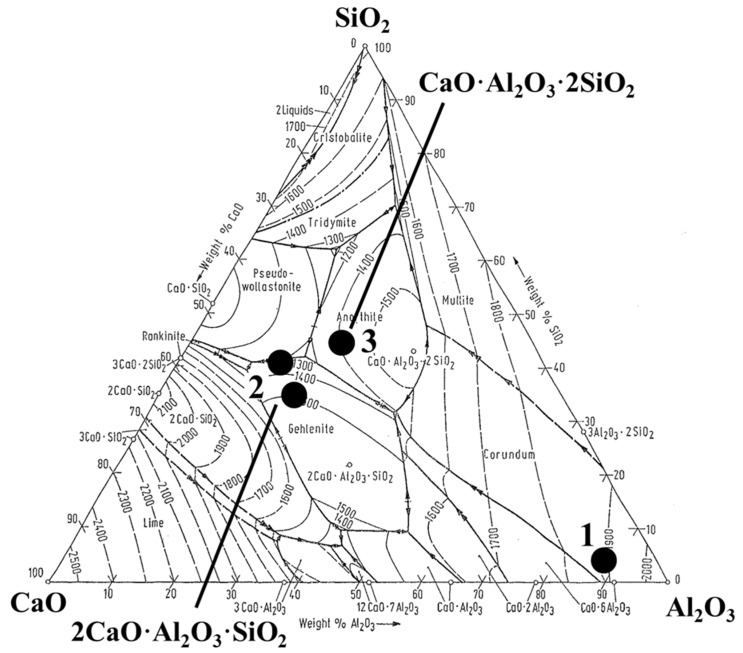
Typical compositions of oxide inclusions observed in experimental trials of AISI 316L stainless steel [42].

**Figure 23:**
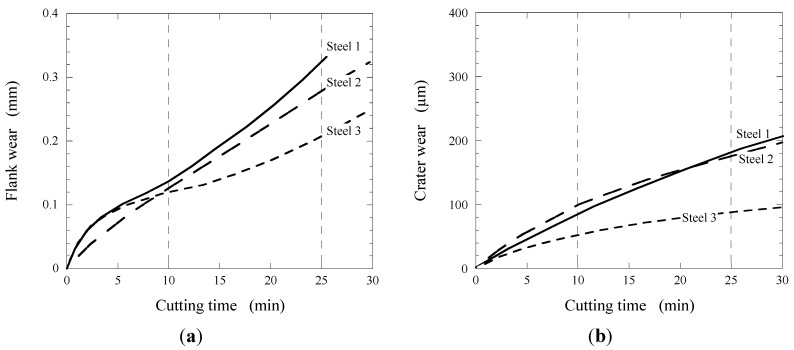
(**a**) Flank wear; and (**b**) crater wear progressions in turning test for AISI 316L steel with different non-metallic inclusions: (1)—alumina (Al_2_O_3_); (2)—gehlenite (2CaO·Al_2_O_3_·SiO_2_); and (3)—anorthite (CaO·Al_2_O_3_·2SiO_2_) [42].

Bletton *et al.* [42] studied the effect of Ca-additions on the composition of oxide inclusions and on the machinability of AISI 316L stainless steel after continuous casting and hot rolling. The main types of oxide and sulfide inclusions in experimental trials of AISI 316L steel are listed in Table 6. The typical compositions of oxide inclusions observed in experimental trials are shown in the CaO-SiO_2_-Al_2_O_3_ ternary phase diagram shown in Figure 22 [42]. The comparative flank and crater wear progressions obtained during machining of these steels are shown in Figure 23a,b.

A cemented carbide cutting tool was used for a conventional turning test using the cutting speed 180 m/min, the feed rate 0.25 mm/rev and the depth of cut 1.5 mm in dry machining. It can be seen that the flank wear (FW) of the tool is approximately similar for all steels, during the initial 10 min of machining. However, the FW values for the Ca-treated steels decreased significantly at a machining time larger than 10 min. For instance, the FW of the Ca-treated steels was 39% (Steel 3) and 15% (Steel 2) smaller in comparison to the reference steel (Steel 1) after 25 min of machining.

**Table 6:** Types of main inclusions in experimental trials of AISI 316L stainless steel [42].

Steel	Ca-Addition	Main Type of Oxide	Main Type of Sulfide
1	No (Ref.)	Alumina, Al_2_O_3_	MnS
2	Yes	Gehlenite, Ca_2_Al[AlSiO_7_]	MnS + (Mn,Ca)S
3	Yes	Anorthite, CaAl_2_Si_2_O_8_	MnS

The flank wear progression of the used cutting tool during machining of Steel 3 which contained anorthite inclusions is about 22% lower after 15 min and 29% lower after 25 min than that of Steel 2 which contained gehlenite inclusions (2CaO·Al_2_O_3_·SiO_2_). For the same setup, the crater wear progression for Steel 3 is about 50% lower in comparison to the Steel 1 and Steel 2 in the range of machining time from 10 to 30 min. This significant improvement of the machinability parameters for Steel 3 can be explained by the lower melting point of anorthite (1400 °C) in comparison to gehlenite (1500 °C) and alumina (1900 °C). A lower melting point means more malleable and softer inclusions, which in turn are less detrimental for the tool wear. The approximate hardness and melting points for some common oxide inclusions in different steels are listed in Table 7 [27]. It can be seen that the Ca-treatment of a liquid steel transform hard Al_2_O_3_ and SiO_2_ inclusions into softer Ca-based inclusions, which have lower melting temperatures. Thus, it is apparent that the anorthite inclusions (CaO·Al_2_O_3_·2SiO_2_) are favorable for machinability.

Kirsch-Racine *et al.* [5] also reported that the Ca-treatment of industrial medium carbon steel (0.35%–0.40% C, 0.02%–0.03% S) promotes a transformation of sharp solid inclusions of Al_2_O_3_ and Al_2_O_3_-MgO into spherical liquid oxides such as CaO-Al_2_O_3_ and 12CaO·7Al_2_O_3_. The compositions of observed inclusions were depended on the amount and yield of the added Ca in the melt. As a result, an increased amount of liquid oxides in the steel from 0% to 52% and 97% promotes an improvement of the turning machinability at high cutting speeds by 21% and 31%, respectively. The authors explained the decreased tool wear by the formation of CaO-Al_2_O_3_ inclusions, which have lower melting temperatures and better visco-plastic properties. This resulted in the formation of a protective/lubrication layer (Built-Up Layer) on the cutting tool.

Moreover, according to the data obtained from the literature review, it was found that calcium-based oxide inclusions have advantages compared to most other oxide inclusions with respect to the mechanical properties as well as machinability.

**Table 7:** Approximate hardness and melting temperature of some common oxides in steels [27].

Inclusion	Inclusion Stoichiometry	Hardness (kg/mm^2^)	Melting Temperature, T_m_ (°C)
Alumina	Al_2_O_3_	3000	2050
Silicate	SiO_2_	1600	1720
Calcium aluminates	(CaO)-(Al_2_O_3_)	930	1330–1839
Gehlenites	Ca_2_Al[AlSiO_7_]	1200	1310–1590
Anorthites	CaAl_2_Si_2_O_8_	850	1170–1550

Ca-treated steels show an improved overall effect on the machinability. It correlates to a decreased power consumption (force, torque), a higher productivity (metal removal rate), a controlled chip breakage, and an increased tool life (decreased tool wear) [25,28,30,32]. Moreover, the improved machinability is often linked to a formation of a so called protective layer [5,28,30,69]. The supporting argument is usually based on SEM analysis of a cutting surface of tool and detection of elements like Ca, Al, O, Mn, S *etc.* This additional protective layer is obtained from components of non-metallic inclusions during mechanical machining of steel. This protective layer is used as an additional lubricant between the surfaces of steel piece and cutting tool during machining. 

Thus, according to the data obtained from literature review, it was found that calcium-based oxide inclusions have advantages compared to most other oxide inclusions, with respect to the mechanical properties as well as the machinability.

## 5. Summary

Based on the literature review, the effects of characteristics (such as composition, morphology, *etc.*) of different non-metallic inclusions on machinability of various steel grades were discussed and summarized. The main mechanisms of steel fracture during different mechanical machining operations, tool wear and behavior of various non-metallic inclusions in a cutting zone and in metal chips were considered. Comparative characteristics of non-metallic inclusions and their effect on some mechanical properties and machinability of different steels were summarized and discussed. Finally, some more effective methods which are commonly used today in steelmaking companies for improvement of machinability of various industrial steel grades, were discussed and compared. This discussion can significantly help to select effective methods for modification and control of non-metallic inclusions in the liquid steel to obtain a desired balance between mechanical properties and machinability of various steel grades.
